# Asian Americans are less willing than other racial groups to participate in health research

**DOI:** 10.1017/cts.2019.372

**Published:** 2019-05-28

**Authors:** Yiyang Liu, Amy Elliott, Hal Strelnick, Sergio Aguilar-Gaxiola, Linda B. Cottler

**Affiliations:** 1 College of Public Health and Health Professions and College of Medicine, University of Florida, Gainesville, FL, USA; 2 Albert Einstein College of Medicine, Bronx, NY, USA; 3 School of Medicine, University of California, Davis, CA, USA

**Keywords:** Asian Americans, translational research, research participation willingness, health research, community engagement, trust

## Abstract

**Background:**

Asian Americans constitute 5% of the U.S. population. Their willingness to participate in research is important to examine because it influences participation rates and the representativeness of study results.

**Methods:**

A total of 17,339 community members participated from six diverse Clinical and Translational Award (CTSA) sites. Community members were asked about their willingness to volunteer for eight different types of health research, their expectation of monetary compensation for research participation, their trust in research and researchers, their preferred language to receive health information, and their socio-demographic background. We examined Asian Americans’ willingness to participate in various types of health research studies and compared their perceptions with other racial/ethnic groups (i.e., Asian *n* = 485; African-American *n* = 9516; Hispanic/Latino *n* = 1889; Caucasian *n* = 4760; and other minority *n* = 689).

**Results:**

Compared to all other racial/ethnic groups, Asian Americans were less willing to participate in all eight types of health research. However, Asian Americans reported a lower amount of fair compensation for research participation than African-Americans and Hispanics/Latinos but were as likely to trust researchers as all other racial/ethnic groups.

**Conclusion:**

Asian Americans are less willing to participate in health research than other racial/ethnic groups, and this difference is not due to dissatisfaction with research compensation or lower trust in researchers. Lack of trust in research and language barriers should be addressed to improve representativeness and generalizability of all populations in research.

## Background

The progress of public health and medicine today results primarily from research studies that involve human participants. Although some progress has been made in reducing the burden of disease among people of racial/ethnic minority groups, most disparities in disease burden remain unchanged and, in fact, are increasing in some areas [1, 2]. Racial/ethnic minorities must be included in all health research studies to ensure equitable participation opportunities and generalizability of findings and guarantee correct estimates of effects in treatment heterogeneity and prevention trials [1,3]. High rates of racial/ethnic minority participation in health research may positively impact advances in health research for underrepresented racial/ethnic minority groups [4].

According to the United States (US) Census, there are 21.4 million Asian Americans in the US [5]. They represent 5% of the US population and are one of the fastest growing racial/ethnic minority populations. Therefore, it is important to include a representative sample of Asian Americans in health research. However, past research has consistently found that Asian Americans have lower research participation rates than other racial/ethnic groups [6–12] and that they are underrepresented in cardiovascular disease research [13], mental health research [14,15], cancer research [16], women’ health research [12] and community-engaged research [17].

Several factors have been associated with low participation rates in racial/ethnic minorities. For example, they may not be given enough opportunities to participate in research, they may be less interested in research participation, they may have more language barriers, may lack trust in research and/or in the consent process, or have higher expectations for research participation compensation than Caucasians [7,18–20]. Some of these factors have not been well-examined among Asian Americans. More research is needed to examine factors that contribute to Asian Americans’ low participation rates and low willingness to participate in health research.

In addition, past findings on differences in willingness to participate in research between Asian Americans and other racial/ethnic groups have focused on studies of reproductive medicine or a specific health condition such as cervical cancer [6,8,10,12]. Willingness to participate in these studies is greatly influenced by an individual’s health status and perceived risks and benefits. One qualitative study found that the leading motivation for participating in research is health care improvement for self or family [4]. Participants with health needs may be more willing to participate in health research when the research is related to a health condition they have [20]. Few studies have examined the differences between Asian Americans and other racial/ethnic groups in willingness to participate in health research by research activities that are not tied to any specific health conditions [7].

The current paper aims to fill this gap in the literature. Using a large multisite, community-based study sample, we compared the willingness to participate in eight different health research types that specified different research activities. In addition, we explored expectations for fair monetary compensation, trust in research and researchers, and language barriers as possible reasons that Asian Americans may have lower willingness to participate in research than other racial/ethnic groups.

## Methods

### Participant Recruitment

A total of 17,339 community members were recruited from six CTSA sites through three study cohorts from 2010 to 2017. The three study cohorts were Sentinel Network phases 1 and 2 and HealthStreet [21]. A community health worker (CHW) model was used to recruit participants at all sites for all three cohorts [7]. All CHWs were trained on the protection of research participants, confidentiality, risk management, making connections with community members, and the meaning of each question. CHWs recruited community members at venues where people usually congregate, such as bus stops, libraries, laundromats, churches, fitness centers, gas stations, and parks [7]. Sentinel Network phase 1 recruited 5979 individuals from 2010 to 2011 with the collaboration of five CTSA sites: Washington University in St. Louis, Missouri; University of Rochester, Rochester, New York; University of Michigan, Ann Arbor, Michigan; Albert Einstein College of Medicine, Bronx, New York; and University of California, Davis (UC Davis), Sacramento, California. In 2012, the second phase of Sentinel Network was implemented. The second phase added the University of Florida (UF), Gainesville, Florida, as a study site and recruited a total of 2371 participants. After the Sentinel Network cohorts closed, UF continued to recruit participants through its ongoing community engagement program, HealthStreet. A total of 8989 individuals were recruited by the end of 2017 through HealthStreet.

Upon obtaining informed consent, CHWs conducted a brief interview with participants, using an IRB-approved Health Intake Assessment. The core set of questions on the Health Intake Assessment included questions on demographics, top health and neighborhood concerns, willingness to participate in research across various types, and self-reported health conditions. This core set of questions was consistently assessed in all study cohorts and across all study sites. Some additional questions were added to the Health Intake Assessment after Sentinel Network phase 1, but the core function of the Health Intake remained the same, which was to assess community members’ health needs and concerns and to link people to medical and social services and opportunities to participate in health research. The interview took approximately 15-30 minutes to complete, and there was no monetary compensation for study participation.

### Measurements

Participants selected their self-identified race from the following choices: American Indian/Alaskan Native, Asian, Black/African-American, Native Hawaiian/Pacific Islander, White, and Other. Ethnicity was asked through the question, “Are you Hispanic or Latino?” (yes/no). In this analysis, race/ethnicity was classified into five groups: Asian American, African-American, Hispanic/Latino, Caucasian, and other minority. Other demographic variables measured included age, sex (female/male), and education level (high school or below/above high school). Participants’ self-reported health conditions were assessed through the question “Have you ever been told you had or have you ever had a problem with [health condition]?” for cancer, heart disease, depression, diabetes (type I or II), and hypertension. These conditions are the top health concerns reported by community members [7]. A new variable was created that counts the number of conditions a participant had, and this variable was used as the proxy of overall health conditions in the multivariate regression models.

Willingness to participate in health research studies was the primary outcome of interest for this analysis. Participants were asked about eight scenarios of health research, elicited by “There are many types of health studies. Would you volunteer for a health research study that [research scenario]?” with yes/no responses. The eight different scenarios were selected by the national Clinical and Translational Science Award (CTSA) Sentinel Network Workgroup and covered the most common types of CTSA health research [7,21]. Participants were asked if they would participate in a study: (1) that only asked questions about their health, (2) that required seeing their medical records, (3) that required giving a blood sample, (4) that required donating a genetic sample, (5) that involved taking medicine, (6) that involved staying overnight in a hospital or clinic, (7) that required using medical equipment, and (8) when they were not paid.

We also identified possible factors contributing to racial/ethnic differences in willingness to participate in health research. Expectation for compensation was asked through the following question, “How much money do you think is a fair amount for participation in a study that lasts about an hour and a half and involves an interview and a blood test?” Participants could answer any value between $0 and $999. Answers beyond $999 or answers indicating research participation was “priceless” were coded as $999. This question was included in the core set of the questions and was asked for all study cohorts. Trust toward health research was assessed with 2 questions, which were added to the Health Intake Assessment in July 2014: “On a scale of 1 to 10, where 1 is “not at all” and 10 is “completely,” how much do you trust research?” And a similar question was asked for trust in researchers. Preferred language was added in 2012 for Sentinel Network phase 2 and was captured by the question “In which language would you like to receive your health information?” and responses were categorized as English or Non-English.

### Analyses

Data analyses were conducted using SAS, version 9.4. All participants were stratified into five racial/ethnic groups (Asian American, African-American, Hispanic/Latino, Caucasian or other minority). Demographic characteristics and health conditions were summarized using descriptive statistics by racial/ethnic group. Chi-square tests were performed to compare whether there were any significant racial/ethnic differences in willingness to participate in the eight different types of health research studies. Multivariate logistic regression was used to calculate adjusted odds ratios (ORs) and 95% confidence intervals for willingness to participate in research for Asian Americans versus other racial/ethnic groups, controlling for the covariates of age, sex, education, health condition, study cohort, and study site. Due to the imbalanced number of participants across racial/ethnic subgroups, a sample weight of 1/sqrt(n) (one divided by the square root of the sample size “n”) was assigned to each racial/ethnic group, where n is the sample size for the subgroup. In addition, Wilcoxon rank sum tests and chi-square tests were used to explore expectation for compensation, trust, and language as possible factors contributing to differences found in willingness to participate in health research among racial/ethnic groups.

## Results

In total, 17,339 individuals were included in the analysis. Participants’ demographic characteristics and their health conditions are described in Table 1. Among the study sample, the mean age was 43.5 years. Asian Americans were younger (mean = 37.2 years) than all other groups. Around 60% of the study sample was female, and this percentage was consistent across all five racial/ethnic groups. Most of the study sample (58.5%) had a high school education or less, 68.9% of Asian Americans achieved education levels beyond high school, which was the highest proportion among the racial/ethnic groups in our sample. The majority of the study sample (56.1%) came from the UF site, followed by the Washington University in St. Louis site (16.1%). Of the 485 Asian American participants, 36.3% came from the UC Davis site, 32.6% came from the University of Michigan site, and 22.1% came from the UF site. A majority (53.8%) of participants reported at least one health condition. The prevalence of cancer, depression, diabetes, and hypertension was lowest among Asian Americans, but the prevalence of heart disease was higher among Asian Americans compared to the other racial/ethnic groups. Overall, Asian Americans had better self-rated health than the other racial/ethnic groups, with only 29.1% reporting any of the queried health conditions—significantly lower than the other groups (*p* < 0.0001).

**Table 1.** Demographic characteristics and health condition of participants by race/ethnicity

	Total^a^ *n* = 17,339	Asian American *n* = 485	African-American *n* = 9516	Hispanic/Latino *n* = 1889	Caucasian *n* = 4760	Other minority *n* = 689	*p* value
Mean age (SD)	43.5 (16.1)	37.2 (17.6)	42.7 (15.4)	41.6 (15.9)	46.8 (16.6)	42.6 (15.5)	<0.0001
	*n* (%)	*n* (%)	*n* (%)	*n* (%)	*n* (%)	*n* (%)	
Sex							0.00012
Male	7082(41.0)	192 (39.8)	4003 (42.2)	701 (37.4)	1902 (40.1)	284 (45.6)	
Female	10185 (59.0)	290 (60.2)	5478 (57.8)	1174 (62.6)	2844 (59.9)	399 (58.4)	
Education							<0.0001
High school or below	100050 (58.5)	147 (31.1)	6298 (66.6)	1164 (63.6)	2098 (44.3)	343 (50.7)	
Above high school	7127 (41.5)	325 (68.9)	3165 (33.4)	665 (36.4)	2639 (55.7)	333 (49.3)	
Study site							<0.0001
Washington	2778 (16.1)	7 (1.4)	2298 (24.2)	38 (2.0)	389 (8.2)	46 (6.7)	
Rochester	1183 (6.8)	8 (1.7)	607 (6.4)	131 (6.9)	369 (7.7)	68 (9.9)	
Michigan	1337 (7.7)	158 (32.6)	411 (4.3)	70 (3.7)	623 (13.1)	75 (10.9)	
Einstein	868 (5.0)	29 (6.0)	243 (2.6)	430 (22.8)	96 (2.0)	73 (10.6)	
UC Davis	1451 (8.4)	176 (36.3)	300 (3.1)	618 (32.7)	253 (5.3)	104 (15.1)	
Florida	9722 (56.1)	107 (22.1)	5657 (59.5)	602 (31.9)	3033 (63.7)	323 (46.9)	
Health condition							
Cancer	1183 (7.0)	16 (3.4)	421 (4.5)	84 (4.6)	609 (13.0)	53 (7.9)	<0.0001
Heart disease	445 (2.6)	19 (3.9)	188 (2.0)	63 (3.4)	147 (3.1)	28 (4.2)	<0.0001
Depression	4699 (27.3)	40 (8.3)	2097 (22.2)	462 (24.7)	1892 (39.9)	208 (30.6)	<0.0001
Diabetes	2255 (13.2)	44 (9.1)	1298 (13.8)	269 (14.4)	544 (11.5)	100 (14.8)	<0.0001
Hypertension	5748 (33.4)	87 (18.0)	3460 (36.6)	522 (27.9)	1468 (31.1)	211 (30.9)	<0.0001
Any health condition	9327 (53.8)	141 (29.1)	4956 (52.1)	902 (47.8)	2955 (62.1)	373 (54.1)	<0.0001

a Among the total 17339 participants, 5979 were recruited from the Sentinel Network 1 cohort, 2371 were recruited from the Sentinel Network 2 cohort, and 8989 were recruited from the HealthStreet cohort.

Willingness to participate in different types of health research among the five racial/ethnic groups is shown in Table 2. The two types of activities that Asian Americans were most willing to participate in were research studies that only asked questions about health (75.6%) and studies that asked for a blood sample (61.7%). Research studies that required taking a medication (30.5%) and studies that asked participants to stay overnight in a hospital or clinic (38.6%) had the lowest rates of Asian Americans reporting willingness to participate. Similar trends could be observed in African-Americans, Hispanics/Latinos, Caucasians, and other minorities but with higher percentages of individuals willing to participate in these studies. There was a significant racial/ethnic difference (*p* < 0.0001) in willingness to participate in all eight activities of health research studies with Asian Americans having the lowest willingness. These differences were further analyzed using multivariate logistic regression.

**Table 2.** Willingness to participate in 8 scenarios of health research by race/ethnicity

	Asian American *n* = 485	African-American *n* = 9516	Hispanic/Latino *n* = 1889	Caucasian *n* = 4760	Other minority *n* = 689	*p*-value
Would participate in a study…	*n* (%)	*n* (%)	*n* (%)	*n* (%)	*n* (%)	
if only asked questions about health	381 (75.6)	8584 (90.2)	1633 (86.5)	4407 (92.6)	591 (85.8)	<0.0001
if researchers wanted to see your medical records	272 (56.1)	7889 (82.9)	1437 (76.1)	3975 (83.5)	506 (73.4)	<0.0001
if you had to give a blood sample	299 (61.7)	7907 (83.1)	1496 (79.2)	4113 (86.4)	531 (77.1)	<0.0001
if you had to give a genetic sample	277 (57.1)	7708 (81.0)	1405 (74.4)	4035 (84.8)	526 (76.3)	<0.0001
if you had to take medicine	149 (30.7)	5462 (57.4)	930 (49.2)	2918 (61.3)	348 (50.5)	<0.0001
if asked to stay overnight in a hospital or clinic	187 (38.6)	6823 (71.7)	1135 (60.1)	3404 (71.5)	463 (65.8)	<0.0001
if you might have to use medical equipment	261 (53.8)	7605 (79.9)	1350 (71.5)	3916 (82.3)	499 (72.4)	<0.0001
if you didn’t get paid	261 (53.8)	6382 (67.1)	1270 (67.2)	3603 (75.7)	466 (67.6)	<0.0001

The ORs from regression models are listed in Table 3, comparing Asian Americans to the other racial/ethnic groups. Age, sex, education level, health condition, study site, and study cohort were controlled in the model. Asian Americans were less willing to participate than all other racial/ethnic groups in all eight types of health research studies. Comparing the four ORs calculated for each study type, the ORs were smallest when comparing Asian Americans to Caucasians, and largest when comparing Asian Americans to African-Americans or to other minorities for all study types. This indicates that the difference in willingness to participate in research between Asian Americans and Caucasians is larger than the difference between Asian Americans and African-Americans or between Asian Americans and other minorities. In addition, the results show that the differences in ORs in willingness to participate in research between Asian American and other racial/ethnic groups was the smallest for studies where participants did not get paid (Asian American vs. African-American: OR=0.74; Asian American vs Hispanic/Latino: OR=0.63; Asian American vs. Caucasian: OR=0.53; and Asian American vs. other racial minority: OR=0.70).

**Table 3.** Multivariate logistic regression with adjusted odds ratio for willingness to participate in different scenarios of health research studies, controlling for age, sex, education level, health condition, study site, and study cohort

	Asian vs. African-American	Asian vs. Hispanic/Latino	Asian vs. Caucasian	Asian vs. Other minority
Would participate in a study…	OR (95%CI)	OR (95%CI)	OR (95%CI)	OR (95%CI)
if only asked questions about health	0.57 (0.47, 0.68)	0.50 (0.41, 0.60)	0.44 (0.36, 0.53)	0.70 (0.57, 0.86)
if researchers wanted to see your medical records	0.61 (0.53, 0.71)	0.49 (0.42, 0.57)	0.44 (0.38, 0.51)	0.72 (0.61, 0.85)
if you had to give a blood sample	0.74 (0.63, 0.86)	0.49 (0.42, 0.58)	0.46 (0.39, 0.53)	0.73 (0.62, 0.87)
if you had to give a genetic sample	0.70 (0.61, 0.82)	0.53 (0.46, 0.62)	0.42 (0.37, 0.49)	0.62 (0.50, 0.73)
if you had to take medicine	0.69 (0.60, 0.80)	0.65 (0.56, 0.76)	0.48 (0.42, 0.56)	0.68 (0.58, 0.80)
if asked to stay overnight in a hospital or clinic	0.51 (0.45, 0.59)	0.49 (0.43, 0.57)	0.46 (0.40, 0.52)	0.48 (0.41, 0.57)
if you might have to use medical equipment	0.57 (0.50, 0.67)	0.50 (0.43, 0.58)	0.43 (0.37, 0.49)	0.62 (0.53, 0.73)
if you didn’t get paid	0.74 (0.64, 0.85)	0.63 (0.54, 0.72)	0.53 (0.46, 0.61)	0.70 (0.60, 0.82)

We then explored expectation of monetary compensation for study participation, trust in research and researchers, and language as possible factors that contribute to these differences (Table 4). For research participation compensation, Asian Americans reported on average that $65 was a fair amount of compensation for a study that involved an interview and a blood test, an amount higher only than what Caucasians expected as fair ($58; the difference is not statistically significant), and significantly lower than the average amount reported by African-Americans ($101, *p* < 0.0001) and Hispanics/Latinos ($83, *p* < 0.05). On a scale of 1 to 10, all racial groups reported an average trust in research and researchers between 6.9 and 7.8. Asian Americans reported statistically lower trust in research than Caucasians and Hispanics/Latinos (*p* < 0.05), but there were no statistical differences in reporting trust in researchers between Asian Americans and individuals of other racial/ethnic groups. Lastly, 87.9% of Asian Americans, compared to over 99% of African-Americans, Caucasians, and other racial/ethnic minorities reported English as their preferred language to receive health information (*p* < 0.0001).

**Table 4.** Exploratory factors to explain the differences in willingness to participate in health research

	Asian American	African-American	Hispanic/ Latino	Caucasian	other minority
	mean (SD), *n*, Q1, Q3	mean (SD), *n*, Q1, Q3	mean (SD), *n*, Q1, Q3	mean (SD), *n*, Q1, Q3	mean (SD), *n*, Q1, Q3
**Expectation for compensation**					
Fair amount compensation ($) for participation in a study that involves an interview and a blood test^a,b^	$65 ($86), 388, $20, $95	$101 ($164), 7893, $30, $100	$83 ($126), 1471, $20, $100	$58 ($97), 4173, $20, $50	$80 ($118), 546, $25, $100
	mean (SD), *n*	mean (SD), *n*	mean (SD), *n*	mean (SD), *n*	mean (SD), *n*
**Trust**^d^					
Trust In research (1 to 10)^c,b^	7.2 (1.8), 47	7.0 (2.1), 2544	7.8 (1.8), 329	7.6 (1.8), 1623	7.5 (2.1), 140
Trust in researchers (1 to 10)	7.4 (1.9), 47	6.9 (2.2), 2536	7.7 (1.9), 329	7.5 (1.9), 1619	7.4 (2.2), 137
	*n* (%)	*n* (%)	*n* (%)	*n* (%)	*n* (%)
**Preferred language to receive health information**^e^					
English	123 (87.9)	6145 (99.9)	679 (78.1)	3296 (99.7)	406 (99.3)
Non-English	17 (12.1)	5 (0.1)	191 (22.0)	9 (0.3)	3 (0.7)

a Wilcoxon rank sum test *p* < 0.0001 between Asian and African-American.

b Wilcoxon rank sum test *p* < 0.05 between Asian and Hispanic/ Latino.

c Wilcoxon rank sum test *p* < 0.05 between Asian and Caucasian.

d Only asked for HealthStreet participants who were recruited after July 2014.

e Only asked for participants recruited after May 2012; paired chi-square test *p* < 0.0001 between Asian, African-American, Caucasian and other minority; paired chi-square test *p* < 0.05 between Asian and Hispanic/Latino.

## Discussion

In this study, we combined three cohorts of community members recruited from six CSTA sites in the US between 2010 and 2017. Among 17,339 community members, a total of 485 Asian Americans were identified. Their willingness to participate in research was compared to the willingness of African-Americans, Hispanic/Latinos, Caucasians, and other minorities. Asian Americans were significantly less willing than any other racial/ethnic group to participate in all eight types of health research.

Past research has found that age, sex, education level, and participants’ health conditions are associated with willingness to participate in research. Some studies have found that older adults are more willing to participate in health research studies [22], while others have found the opposite [23]. Many studies have found females to be more likely to participate in health research than their male counterparts [23–25]. Higher education level has also found to be associated with higher willingness to participate in research [22,23]. Lastly, as we mentioned previously, people’s health conditions may also influence their willingness to participate in health research, as they are interested in studies related to their illnesses. [20]. In our study, Asian Americans were younger, had higher education levels, and fewer health conditions. But even after controlling for these factors, Asian Americans were less willing to participant in all types of health research than all other racial/ethnic groups. Asian American’s greater educational achievement and lower willingness to participate contradicts past findings. Exploratory analyses were conducted to explain these differences. Financial compensation could increase willingness to participate in research, and it is often used in study recruitment to encourage participation [26–28]. If the financial compensation offered by the researcher is less than the expected fair amount of payment for study participation, participants may be less motivated and less willing to join the research. In our analysis, although Asian Americans were least willing to participate in unpaid research, the difference for this type of study was the smallest. In addition, when we asked participants how much they thought was a fair amount of compensation for participating in a study that involved a 1.5-h long interview and a blood test, Asian Americans reported $65 as a fair compensation. This amount was the second lowest among all five racial/ethnic groups: only $6 more than Caucasians (not statistically significant), but $35 less than African-Americans (statistically lower, *p* < 0.0001), $18 less than Hispanics/Latinos (statistically lower, *p* < 0.05) and $16 less than other minorities (not statistically significant). Therefore, we believe satisfaction with study compensation is not likely to be a major contributing factor in the difference we found in willingness to participate in research between Asian Americans and other racial/ethnic groups.

Trust levels in research and in researchers were also assessed (trust measures come only from the HealthStreet cohort). Several studies have found mistrust in health research to be a key barrier in preventing racial/ethnic minority populations from participating in research [20,29]. This effect is especially strong in explaining the willingness to participate in research difference between Caucasians and African-Americans [18,20,30,31]. One systematic review found that concerns behind the mistrust were different between racial/ethnic groups [18]. In contrast with the mistrust among African-Americans due to the perception that research will only benefit Caucasians or the research institution, but not participants of color, Asian Americans’ lack of trust may be due to concerns about signing consent as they perceive it as a way of relinquishing their rights [18].

In our analyses, we found African-Americans to have the lowest trust in research and researchers. This is consistent with past literature. In addition, we found a modest statistical difference in trust in research between Asian Americans (7.2) and Hispanics/Latinos (7.8) and between Asian Americans and Caucasians (7.6) (*p* < 0.05). No statistical differences were observed when measuring trust in researchers: trust reported by Asian Americans (7.4) was very close to what was reported by Caucasians (7.5) and other minorities (7.4). Building a trust relationship is important to reduce the differences in willingness to participate in research across race/ethnic groups.

There are important implications borne out of the current analyses. We found that Asian Americans were less willing to participate in each and all of eight types of health research. In order to recruit a diverse study sample and ensure the representativeness of the results, investigators may need to invest additional time and resources to enroll adequate numbers of Asian American participants successfully. One possible solution would be to choose appropriate venues for sampling, including venues where Asian Americans congregate, such as at local Asian supermarkets, faith-based organizations, Asian community centers, and non-profit organizations serving the Asian American populations. At those venues, recruitment teams could reach out to a large number of Asian Americans in a short period of time. Moreover, recruiting at those places could help improve engagement and trust of the Asian American community as a whole, spread information about health research through word of mouth, and make Asian Americans feel more respected and wanted in research studies [18,32]. In addition, having a diverse research team will also increase trust in the research enterprise.

Findings on participants’ willingness to participate in research with no payment and their self-reported fair compensation for study participation indicates that increasing monetary compensation may not be an effective way to improve willingness to participate relevant to the Asian American population. In addition, we found that a lack of trust in research may contribute to why Asian Americans were less willing to participate in health research than Caucasians and Hispanics/Latinos. However, this factor does not explain the difference between Asian Americans and African-Americans, since Asian Americans reported greater trust in both research and researchers compared to African-Americans.

We also suspect that culture and language barriers may contribute to the lower willingness to participate in health research in Asian Americans [18–20]. In our analysis, we used preferred language to receive health information as a proxy for possible language barriers and found that a lower percentage of Asian Americans preferred English as the language to receive health information than African-Americans, Caucasians and other minorities. It is noteworthy that the language barrier was present for both Asian Americans and Hispanics/Latinos and may be even more so for Hispanics/Latinos. Language barriers may not be a potential explanation for the difference between Asian Americans and Hispanics/Latinos in research participation willingness. Cultural barriers were not assessed in this study, but other studies have found that culture and language barriers are associated with a lack of confidence [18], the feeling of not belonging to the community [33], and low knowledge about health research [34] which may in turn, contribute to lower willingness to participate in research among Asian Americans. Advertising the study using culturally and linguistically relevant recruitment materials [35], increasing the number of Asian American CHWs and including Asian American researchers as part of the recruitment team [18] may help buffer the language and cultural barriers and increase Asian Americans’ participation in research.

These analyses have a few limitations. First, individuals who are unwilling to participate in any research may also have refused to join our HealthStreet needs assessment study in the first place, which may lead to selection bias and underrepresentation of people who do not trust research/researchers. In our sample, Asian Americans were younger and had a higher educational level than other racial/ethnic groups, and these factors are usually associated with a greater willingness in research participation. Further, participants must have been able to interact with the CHWs in English to be included in the study. The differences in preferred language may be a function of the convenience sample used. Moreover, the results may not be generalizable to health research that does not require participants to speak English. Second, although the primary aim of the current analysis was to assess the differences in willingness to participate in health research between Asian Americans and other racial/ethnic groups, we extended our analyses to explore several reasons that may contribute to differences in willingness to participate in research. The two questions on trust in research and researchers were not added to the Health Intake Assessment until July 2014 and only for the HealthStreet cohort. The results from these two questions may be regional instead of representing all Sentinel Network sites. In addition, since the purpose of the Health Intake Assessment was not to systematically examine willingness to participate in research, but instead to understand community needs and navigate participants to health research and services, many factors that may contribute to differences in willingness to participate in research were not assessed. Similarly, our Health Intake Assessment did not distinguish among the wide diverse national origins and languages within the Asian American population.

Future studies should consider other factors, such as cultural and language barriers, political climate, fear, stigma, lack of time, and experience with the health care system, etc., as possible contributors to differences in willingness to participate in research [18].

## Conclusion

The current analysis used a large multisite community sample and found that Asian Americans are less willing to participate in all eight types of health research compared to other racial/ethnic groups. We highlight that trust is a major factor that contributes to willingness to participate in research among everyone, but especially Asian Americans.

## References

[1] Yancey AK , Ortega AN , Kumanyika SK. Effective recruitment and retention of minority research participants. Annual Review of Public Health 2006; 27: 1–28. 10.1146/annurev.publhealth.27.021405.102113 16533107

[2] Satcher D , et al. What if we were equal? A comparison of the black-white mortality gap in 1960 and 2000. Health Affairs (Millwood) 2005; 24(2): 459–464. 10.1377/hlthaff.24.2.459 15757931

[3] Bishop WP , et al. Effectiveness of a community research registry to recruit minority and underserved adults for health research. Clinical and Translational Science 2015; 8(1): 82–84. 25354322 10.1111/cts.12231 PMC4329024

[4] Bouida W , et al. Willingness to participate in health research: Tunisian survey. BMC Medical Ethics 2016; 17(1): 47. 27492385 10.1186/s12910-016-0131-3 PMC4973371

[5] The United States Census Bureau. Asian-American and Pacific Islander Heritage 2018.

[6] Crider KS , et al. Racial and ethnic disparity in participation in DNA collection at the Atlanta site of the National Birth Defects Prevention Study. American Journal of Epidemiology 2006; 164(8): 805–812. 16877537 10.1093/aje/kwj264

[7] Cottler LB , et al. Community needs, concerns, and perceptions about health research: Findings from the clinical and translational science award sentinel network. American Journal of Public Health 2013; 103(9): 1685–1692. 23409875 10.2105/AJPH.2012.300941 PMC3966684

[8] Johnstone E , et al. Asian women are less likely to express interest in infertility research. Fertility and Sterility 2010; 94(4): 1249–1253. 19800619 10.1016/j.fertnstert.2009.08.011

[9] Sharma H , et al. Asian immigrants to the United States are less likely to donate cryopreserved embryos for research use. Fertility and Sterility 2011; 95(5): 1672–1676. 21329918 10.1016/j.fertnstert.2011.01.123

[10] Giarelli E , et al. Research participation among Asian American women at risk for cervical cancer: Exploratory pilot of barriers and enhancers. Journal of Immigrant and Minority Health 2011; 13(6): 1055–1068. 21512747 10.1007/s10903-011-9461-x PMC5470035

[11] Ma GX , et al. Increasing Asian American participation in clinical trials by addressing community concerns. Clinical Trials 2014; 11(3): 328–335. 24603005 10.1177/1740774514522561 PMC4156927

[12] Talaulikar VS , et al. Low participation rates amongst Asian women: Implications for research in reproductive medicine. European Journal of Obstetrics & Gynecology and Reproductive Biology 2014; 174: 1–4. 24368021 10.1016/j.ejogrb.2013.11.026

[13] Minocher Homji RS , Lakhoo S , Ray JG. Recruitment of immigrant and ethnic minorities in primary prevention trials of cardiovascular disease. QJM: An International Journal of Medicine 2011; 104(6): 469–476. 21398665 10.1093/qjmed/hcr027

[14] Mendoza DB , et al. Minority inclusion in randomized clinical trials of panic disorder. Journal of Anxiety Disorders 2012; 26(5): 574–582. 22445317 10.1016/j.janxdis.2012.02.011

[15] Williams M , et al. Minority participation in randomized controlled trials for obsessive-compulsive disorder. Journal of Anxiety Disorders 2010; 24(2): 171–177. 20143498 10.1016/j.janxdis.2009.11.004 PMC3431431

[16] Pinsky PF , et al. Enrollment of racial and ethnic minorities in the Prostate, Lung, Colorectal and Ovarian Cancer screening trial. Journal of the National Medical Association 2008; 100(3): 291–298. 18390022 10.1016/s0027-9684(15)31241-4

[17] De las Nueces D , et al. A systematic review of community-based participatory research to enhance clinical trials in racial and ethnic minority groups. Health Services Research 2012; 47(3 Pt 2): 1363–1386. 22353031 10.1111/j.1475-6773.2012.01386.x PMC3418827

[18] George S , Duran N , Norris K. A systematic review of barriers and facilitators to minority research participation among African Americans, Latinos, Asian Americans, and Pacific Islanders. American Journal of Public Health 2014; 104(2): e16–e31. 10.2105/AJPH.2013.301706 PMC3935672 24328648

[19] Chang TE , et al. Association of race, ethnicity and language with participation in mental health research among adult patients in primary care. Journal of Immigrant and Minority Health 2015; 17(6): 1660–1669. 25398517 10.1007/s10903-014-0130-8

[20] Slegers C , et al. Why do people participate in epidemiological research? Journal of Bioethical Inquiry 2015; 12(2): 227–237. 25672617 10.1007/s11673-015-9611-2

[21] Cottler LB , Nagarajan R. Real-time assessment of community health needs and concerns. Science Translational Medicine 2012; 4(119): 119mr2. 10.1126/scitranslmed.3003367 22301551

[22] Gao W , et al. Factors associated with willingness to participate in biospecimen research among Chinese Americans. Biopreservation and Biobanking 2014; 12(2): 131–138. 24749880 10.1089/bio.2013.0081 PMC3995351

[23] Heerman WJ , et al. Willingness to participate in weight-related research as reported by patients in PCORnet clinical data research networks. BMC Obesity 2018; 5: 10. 29507737 10.1186/s40608-018-0187-3 PMC5831204

[24] Galea S , Tracy M. Participation rates in epidemiologic studies. Annals of Epidemiology 2007; 17(9): 643–653. 17553702 10.1016/j.annepidem.2007.03.013

[25] Meiklejohn J , Connor J , Kypri K. The effect of low survey response rates on estimates of alcohol consumption in a general population survey. PLoS One 2012; 7(4): e35527. 22532858 10.1371/journal.pone.0035527 PMC3332039

[26] Cook C , Mack J , Cottler LB. Research participation, trust, and fair compensation among people living with and without HIV in Florida. AIDS Care 2018; 30(1): 27–31. 28662587 10.1080/09540121.2017.1338656 PMC5858187

[27] Permuth-Wey J , Borenstein AR. Financial remuneration for clinical and behavioral research participation: ethical and practical considerations. Annals of Epidemiology 2009; 19(4): 280–285. 19230712 10.1016/j.annepidem.2009.01.004

[28] Fry CL , et al. Paying research participants: A study of current practices in Australia. Journal of Medical Ethics 2005; 31(9): 542–547. 16131558 10.1136/jme.2004.009290 PMC1734228

[29] Holzer JK , Ellis L , Merritt MW. Why we need community engagement in medical research. Journal of Investigative Medicine 2014; 62(6): 851–855. 24979468 10.1097/JIM.0000000000000097 PMC4108547

[30] Strekalova YA. When trust is not enough: A serial mediation model explaining the effect of race identity, ehealth information efficacy, and information behavior on intention to participate in clinical research. Health Education & Behavior 2018; 45(6): 1036–1042. 29478354 10.1177/1090198118757822 PMC6572720

[31] Shavers VL , Lynch CF , Burmeister LF. Racial differences in factors that influence the willingness to participate in medical research studies. Annals of Epidemiology 2002; 12(4): 248–256. 11988413 10.1016/s1047-2797(01)00265-4

[32] Chao SZ , et al. Recruitment of Chinese American elders into dementia research: The UCSF ADRC experience. Gerontologist 2011; 51(Suppl 1): S125–33. 21565814 10.1093/geront/gnr033 PMC3092979

[33] Hussain-Gambles M , et al. Involving South Asian patients in clinical trials. Health Technology Assessment 2004; 8(42): iii, 1–109. 10.3310/hta8420 15488164

[34] Tu SP , et al. Clinical trials: Understanding and perceptions of female Chinese-American cancer patients. Cancer 2005; 104(12 Suppl): 2999–3005. 16247796 10.1002/cncr.21524 PMC1810650

[35] Leader AE , et al. Exploring Asian Indian and Pakistani views about cancer and participation in cancer genetics research: Toward the development of a community genetics intervention. Journal of Community Genetics 2018; 9(1): 27–35. 28660599 10.1007/s12687-017-0312-x PMC5752649

